# Formation and evolution of a pair of collisionless shocks in counter-streaming flows

**DOI:** 10.1038/srep42915

**Published:** 2017-03-07

**Authors:** Dawei Yuan, Yutong Li, Meng Liu, Jiayong Zhong, Baojun Zhu, Yanfei Li, Huigang Wei, Bo Han, Xiaoxing Pei, Jiarui Zhao, Fang Li, Zhe Zhang, Guiyun Liang, Feilu Wang, Suming Weng, Yingjun Li, Shaoen Jiang, Kai Du, Yongkun Ding, Baoqiang Zhu, Jianqiang Zhu, Gang Zhao, Jie Zhang

**Affiliations:** 1 Key Laboratory of Optical Astronomy, National Astronomical Observatories, Chinese Academy of Sciences, Beijing 100012, China; 2 National Laboratory for Condensed Matter Physics, Institute of Physics, Chinese Academy of Sciences, Beijing 100190, China; 3 Collaborative Innovation Center of IFSA (CICIFSA), Shanghai Jiao Tong University, Shanghai 200240, China; 4 Key Laboratory for Laser Plasmas (MoE), Department of Physics and Astronomy, Shanghai Jiao Tong University, Shanghai 200240, China; 5 Department of Astronomy, Beijing Normal University, Beijing 100875, China; 6 State Key Laboratory for Geomechanics and Deep Underground Engineering, China University of Mining and Technology, Beijing 100083, China; 7 Research Center for Laser Fusion, China Academy of Engineering Physics, Mianyang 621900, China; 8 National Laboratory on High Power Laser and Physics, Chinese Academy of Sciences, Shanghai 201800, China; 9 School of Physical Sciences, University of Chinese Academy of Sciences, Beijing 100049, China

## Abstract

A pair of collisionless shocks that propagate in the opposite directions are firstly observed in the interactions of laser-produced counter-streaming flows. The flows are generated by irradiating a pair of opposing copper foils with eight laser beams at the Shenguang-II (SG-II) laser facility. The experimental results indicate that the excited shocks are collisionless and electrostatic, in good agreement with the theoretical model of electrostatic shock. The particle-in-cell (PIC) simulations verify that a strong electrostatic field growing from the interaction region contributes to the shocks formation. The evolution is driven by the thermal pressure gradient between the upstream and the downstream. Theoretical analysis indicates that the strength of the shocks is enhanced with the decreasing density ratio during both flows interpenetration. The positive feedback can offset the shock decay process. This is probable the main reason why the electrostatic shocks can keep stable for a longer time in our experiment.

Collisionless shocks (CSs) are ubiquitous in the astrophysical phenomena and mainly occur in the interactions of counter-streaming flows, such as explosive ejecta from supernova sweeping up the interstellar media^1^,^2^, and solar wind passing through the ambient medium^3^,^4^. Since the ion-ion free paths (MFPs) are much larger than the transition width of the shocks, generally, those shocks are excited by the electrostatic force^5^,^6^ and/or Lorentz force^7^,^8^ instead of the Coulomb collisions. Due to the difficulty in directly exploring the underlying microphysics of shock formation in the astrophysical conditions, laboratory experiments can closely study it by creating a scaled-down and controllable system.

Counter-streaming flow (CF) system is a particularly appealing test-bed for studying CSs formation in laboratory. Generally, it can be generated by two methods^9^. One method is to use laser beams ablating a foil to blow out an incoming flow, and the reverse-flow is produced by the scattered light and X-ray from the laser-ablated target^10^,^11^,^12^,^13^. In this case, both flows are generated with the different densities and temperatures. Theoretical study and PIC simulation show that such the distributions of the density and the temperature between both flows can enhance the electrostatic shock (ES) formation process (high Mach number shock generation)^14^,^15^,^16^. Recently, many experiments have reported that a high Mach number (M > 10) ES is produced^12^ and propagates from the downstream to the upstream^13^. The other method is to use two bunches of laser beams simultaneously ablating the facing surfaces of two foils to directly generate the counter-streaming flows. Theoretical analysis indicates that two shocks would form in the interaction region and oppositely propagate into the upstream region^17^,^18^. However, no experiment results are reported until now as far as we know.

Here we report the formation and evolution of a pair of shocks observed in laser-produced counter-streaming flows for the first time in laboratory. Initially, the overlapped shocks (shock with two fronts) form in the interpenetration region (unstable region). Then, these two shocks separate and propagate towards ±*x* directions. The shock transition width is measured in the range of 450–700 μm, in good agreement with the estimated value with the theory of collisionless electrostatic shock^19^. The particle-in-cell (PIC) simulations have been performed and found that a strong electrostatic potential growing from the interpenetration region traps the upstream ions to mediate the shocks formation and reflects some ions back into the upstream. The trapped-ions arriving at the downstream are heated when they cross the shock fronts. Consequently, a temperature gradient between the upstream and the downstream is established. The evolution of shocks is mainly caused by the temperature gradient.

## Experiment results

**Figure 1:**
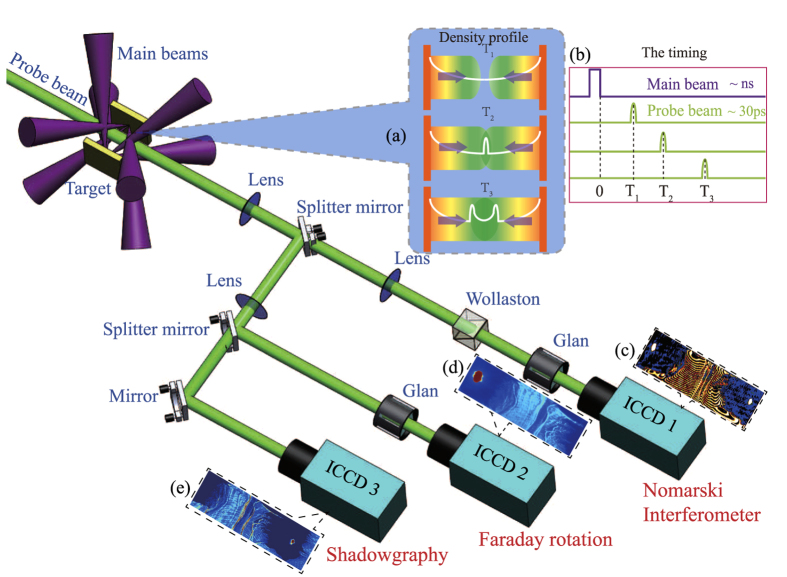
Schematic view of the experimental setup. The CF system is generated by irradiating a pair of opposing copper (Cu) foils with two bunch laser beams (four beams for each bunch). The probe beam passing through the interaction region are recorded by the Nomarski interferometer, Faraday rotation and shadowgraphy. The insets (**a**) illustrate a schematic view of the evolution: (Top) two plasma flows approach to each other, (Middle) after interpenetration, the overlapped region turns unstable and forms a shock, (Bottom) a pair of shocks propagate in opposite directions. This evolution is obtained by changing the delay time between the main beam and the probe beam. The timing is shown in the inset (**b**). The insets (**c**)–(**e**) show the original data of a pair of shocks forms at 10 ns.

Our experiment was performed at the Shenguang II (SG-II) laser facility at the National Laboratory on High Power Lasers and Physics, which can deliver a total energy of 2.0 kJ in 1 ns at 3ω (351 nm). The experimental setup is shown in Fig. 1 and more details are described in the Methods.

**Figure 2:**
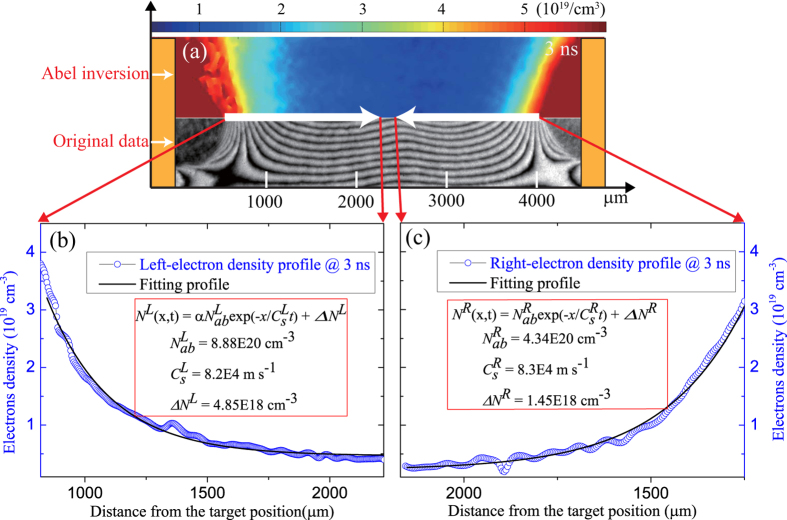
Experimental results before shocks formation. (**a**) The raw image (below) and corresponding electron density map (up) obtained by the Abel inversion before shocks formation at 3 ns. The color bar stands for the value of electrons density. (**b**) and (**c**) are the electron density profile (blue circle) and the corresponding fitting curve (black solid) plotted in the flow direction.

Initial parameters of each flow are important for properties of the generated shocks. For instance, higher flow velocities (~100–1000 km/s) and lower flow densities (~10^18^–10^19^ cm^−3^) can lead to the formation of collisionless electrostatic shock^20^, while the differences of the initial densities and temperatures between both flows can enhance the strength of generated shock (larger Mach number)^14^. Figure 2 (a) shows a typical raw interferogram (below) of CF and the corresponding Abel inversion map (up) before shock formation at 3 ns. The crimson area in the Abel inversion map stands for the plasma density higher than the critical density of the probe beam (~4 × 10^21^ cm^−3^), which corresponds to the no-fringe area in the raw image. No shifted-fringes at the central region indicate that both flows from the opposite target foils have not met with each other. Therefore, the relative velocity of the two flows should be less than 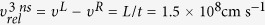. The evolutionary process of flow can be regarded as quasi-isothermal free expansion, which is often treated in such ns-level and kJ-level laser-plasma interaction. The plasma density distribution within each flow is relevant to the sound velocity (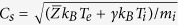), whose density profile complies with the exponential distribution^21^, 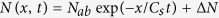. Here *N*_*ab*_ = α*N*_*cr*_ is the ablation density, depending on the parameters of the incident laser, the *N*_*cr*_ = 8.9 × 10^21^ cm^−3^ is the critical density of the driven lasers, *C*_*s*_ is the sound velocity, Δ*N* is density compensation value, *x* is the position and *t* is the time. Figure 2(b) and (c) show the electron density profiles along the central axis obtained by the Abel inversion and the corresponding fitting results. Both flows have the different density distributions with small fluctuations. They share the sound velocity of (8.2–8.3) × 10^4^ m s^−1^.The electron temperature can be roughly estimated as 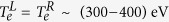 and 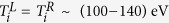 under the assumption of a temperature ratio of about three times between the electrons and the ions in the laser plasma flow, which is commonly observed in the similar experiment^22^,^23^.

**Figure 3:**
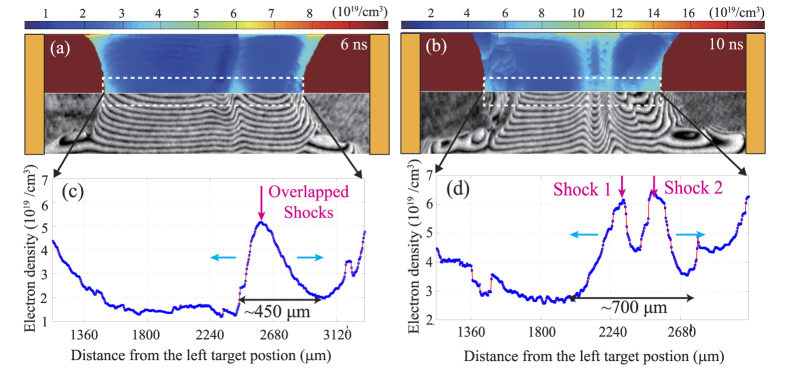
The typical interferogram of shocks formation and evolution. (**a**) and (**b**) are the raw data (below) and electron density distribution maps (up) obtained by the Abel inversion, taken at 6 ns and 10 ns. The color bar stands for the value of electrons density. (**c**) and (**d**) are the corresponding electron density profile plotted along the flow direction. The pink arrows and blue arrows represent the shocks position and the propagation directions, respectively.

Figure 3 shows the typical shock formation and evolution at delay time of 6 ns and 10 ns. At 6 ns (Fig. 3a), a clear abrupt area with shifted fringes appears in the interaction region. This implies that the CF system becomes unstable and the electron densities are redistributed. From the Abel inversion result in Fig. 3(c), the electron density (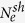) of the peak at *x *= 2560 μm is 5.2 × 10^19^ cm^−3^. According to the fitting expression in Fig. 2(b) and (c),the anticipated overlap electron density from both free flows should be 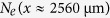

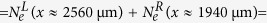
 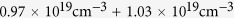 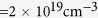, much lower than the observed peak density. Such a large density jump from 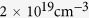 to 5.2 × 10^19^ cm^−3^ indicates shock formation. The appearance of both sharp edges indicates that both overlapped shocks are generated in the interaction region. The shock transition width is about 450 μm. The relative velocity of each flow should be larger than 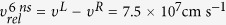. At 10 ns (Fig. 3b), two abrupt areas with shifted fringes are presented in the interaction region. This indicates that the overlapped shocks separate and propagate in the opposite directions. The peak densities (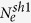 and 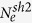) are 6.2 × 10^19^ cm^−3^ and 6.5 × 10^19^ cm^−3^, respectively. The total transition width of the evolving shocks has increased to be about 700 μm. The average shock velocity can be also estimated as 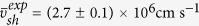, assuming that both shocks are symmetrically moving in Fig. 3(b). The corresponding Mach number is 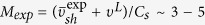, where 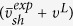 stands for the shock velocity in the upstream frame.

**Figure 4:**
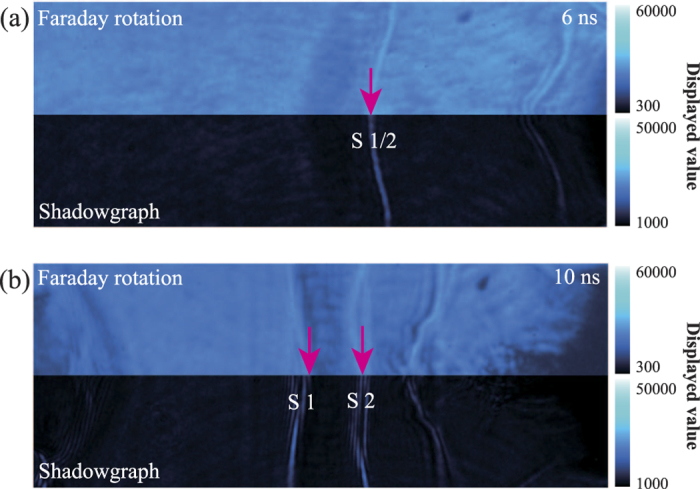
The typical shadowgraph and Faraday rotation of shocks formation and evolution. (**a**) and (**b**) are the corresponding shadowgraph and Faraday rotation image taken at 6 ns and 10 ns. The color bar stands for the intensity of probe beam. In order to distinguish the shadowgraph and Faraday rotation, we have manually adjusted the color bar. The pink arrows represent the shocks position.

Figure 4 shows the corresponding shadowgraph (below) and Faraday rotation image (up) at delay time of 6 ns and 10 ns. The shadowgraph is sensitive to the second derivative of the refractive index (density) of the plasma. Therefore, the presence of the sharp brightness structure in the overlap region represents a large density jump (shock) formation. It is consistent with the observed in the corresponding interferogram of Fig. 3(a) and (b). The Faraday rotation is sensitive to the magnetic field. When a polarized probe beam passes through the magnetized plasma, the polarization will rotate and then cause the intensity change of the probe. Comparing both images in our experiment, no obvious intensity change of the probe beam is observed. It indicates that no magnetic field is excited when counter-streaming flows interact with each other.

The MFPs, as a basic parameter for determining characteristic of the collisionless shocks, can be written in Gaussian units as^24^, 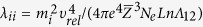, where *m*_*i*_ = *Am*_*p*_ is the ion mass, *e* is the electric charge, 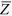 is the average ionization state, *n*_*e*_ is the electron density of each flow and 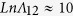 is the Coulomb logarithm. The relative velocity of each flow is 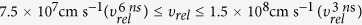, and the electron density of each flow is 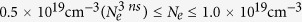
(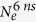). The average ionization state 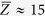 is roughly estimated by a steady-state model^25^, which is mainly determined by the electron temperature *T*_*e*_. Taking those values into above equation, the MFPs are estimated as 16 mm ≤ λ_*ii*_ ≤ 520 mm. Since the MFP is much larger than the width transition region (~450–700 μm), the shocks formed in the CF system are essentially collisionless. In addition, the observed features of the shocks are also different with the collisional case^26^,^27^, where the structure is typically irregular and chaotic rather than well-organized.

It’s well known that two types of collisionless shock can be excited in the CF system. One is electrostatic shock and the other one is electromagnetic Weibel-mediated shock. If the excited shock in the experiment was magnetized, the width of the shock would be order of 100*c*/*ω*_*pi*_ ≈ 15 mm according to previous PIC simulation results^28^, which is much larger than our target separation (*L* = 4.5 mm). For the electrostatic shock, the width of the shock transition region can be estimated as^19^ 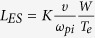, where 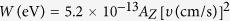 is the kinetic energy, *ω*_*pi*_ is the ion plasma frequency, *T*_*e*_ is the electron temperature, and *K* ~ 30 is a numerical factor implying that the interaction region should be large enough for the electrostatic instability to fully develop. Taking the typical parameters at 6 ns, 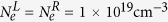, 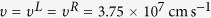, *A*_*Z*_ = 64, 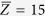, and 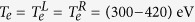 into above equation, we obtained *L*_*ES*_ = (600−800) μm, which is consistent with the value obtained in our experiment.

According to the experimental observation and the theoretical estimation, we can rule out the possibility of magnetized shocks formation in our experiment. Recently, a series of experiments using the kJ-level laser facilities also demonstrate that the self-generated electromagnetic field induced by Weibel-type instabilities cannot support the electromagnetic shock formation, because of the longer shocks formation time at low fluid velocity^29^,^30^,^31^. Therefore, it is reasonable to regard the collisionless shocks in our experiment to be electrostatic.

## Simulation results

**Figure 5:**
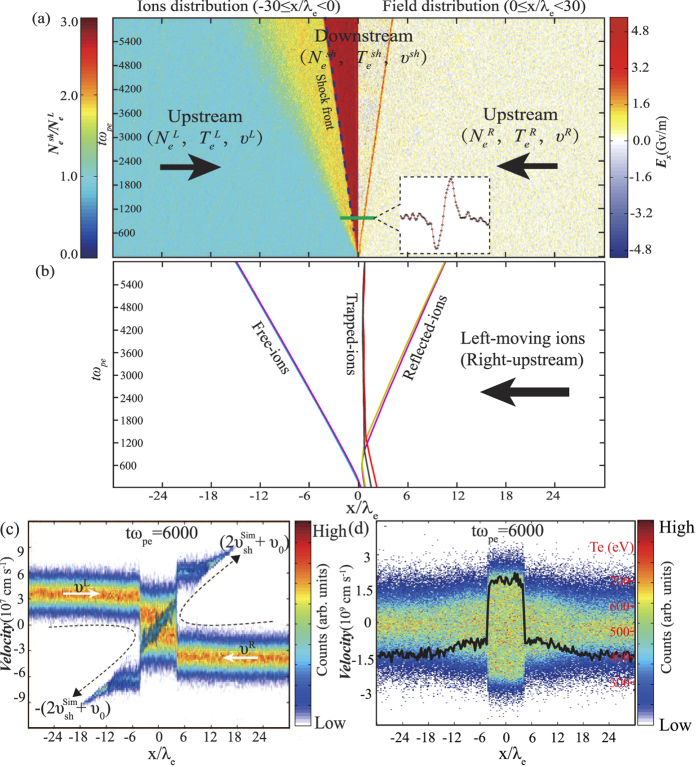
Simulation results. (**a**) The spatio-temporal evolution of the electrostatic shock obtained by our PIC simulation. The left-panel shows the ions distribution and the right-panel show the electrostatic field distribution. The color bar on the left-side is the ions density normalized to the initial ions density. The color bar on the right-side is the strength of the electrostatic field. The blue-dash-line represents the shock front. The inset is the bipolar electrostatic field distribution obtained at *tω*_*pe*_ = 1000 (**b**) The typical ion trajectories for free-ions, trapped-ions and reflected-ions. (**c**) The ions phase-space at *tω*_*pe*_ = 6000. The monoenergetic protons are generated by the electrostatic shocks. (**d**) The electrons phase-space at *tω*_*pe*_ = 6000. The overlapped black line shows the electron temperature distribution.

Figure 5(a) shows the spatio-temporal evolution of electrostatic shock obtained by the PIC simulation (see Methods for the detailed simulation parameters). The image consists of two panels. The left-panel shows the evolution of ions density distribution in the domain 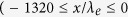) and the right-panel shows the evolution of electrostatic field distribution in the domain (0 ≤ *x*/ *λ*_*e*_ ≤ 1320). The electrostatic field (*E*_*x*_) grows from the overlap region during both flows penetrating each other. After about *tω*_*pe*_ = 1200 (shock formation time), the value of growing field becomes larger than 2.4 Gv/m. The corresponding minimum potential energy can be estimated as 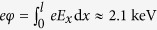, where *l* ≈ *λ*_*e*_ = c/*ω*_*pe*_ = 1.7 μm is the width of the field. The typical kinetic energy of the incoming ions in the shock frame can be estimated as 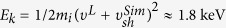 (Here 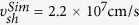 is the shock velocity obtained in the simulation, which keeps constant and is independent of the initial value of flow velocity.), smaller than the potential energy. Obviously, the incoming ions will be slowed down by the potential, accumulated in the overlap region and lead to shock formation. The maximum value of the increasing density is about 2.7, larger than the anticipated factor of two. The evolution of shock front (marked by the blue-dash-line) in the left-panel is well agreement with that of the electrostatic field in the right-panel. It indicates that the electrostatic field excites the shocks formation.

The entire interaction region in Fig. 5(a) consists of downstream (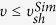) and upstream (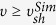), which are separated by the shock front. The region trapping the incoming ions by the potential is the downstream and the rest region (on both sides) is the upstream. To capture the ion motion during the shock formation, the typical ion trajectories are displayed in the Fig. 5(b). The incoming ions from the right-upstream (left-moving ions) are divided into three cases: (i) the trapped-ions are located at the downstream; (ii) the free-ions can freely pass through the downstream and arrive at the opposite upstream; and (iii) the reflected-ions are accelerated back into the upstream. The partial region of the upstream is disturbed by the reflected-ions and the free-ions, which is marked as the yellow area. Figure 5(c) shows the typical phase-space plot of ions at *tω*_*pe*_ = 6000. Some incoming ions are reflected back (accelerated) into the upstream by the strong shock, with a velocity of 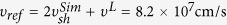. The quasi-monoenergetic protons could be generated by this acceleration mechanism (electrostatic shock acceleration), which has been obtained in the experiment^32^. Figure 5(d) shows the typical phase-space plot of electrons at *tω*_*pe*_ = 6000. The incoming electrons accelerated into the downstream by the field frequently collide with each other and form a thermalized Maxwell-distribution.

## Discussions

When the high-velocity upstream pass through the shock front enters into the downstream, the bulk kinetic energy will be converted into the thermal energy. Consequently, the downstream becomes to be a high temperature region, which has been observed in previous experiment^22^ and simulation^10^. Therefore, the shock front in the shock frame can be regarded as a sharp separation of a thermal pressure (*n*_*e*_*kT*_*e*_) dominant downstream from the ram pressure (*ρυ*^2^) dominant upstream^33^. Figure 5(d) shows the electron temperature distribution crossing the shock fronts. The electron temperature in the downstream is about 700 eV, larger than the initial temperature in the upstream. Obviously, the shock is not in thermal equilibrium state and cannot be stationary. It would evolve at the expense of thermal energy within the downstream. The typical ion sound velocity in the downstream can be estimated as 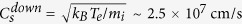 (The ion temperature is negligible in the simulated time scale), which is similar to the shock velocity 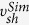.The relationship (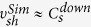) indicates that the shock evolution is mainly driven by the temperature gradient (thermal pressure gradient) between the downstream and the upstream.

**Figure 6:**
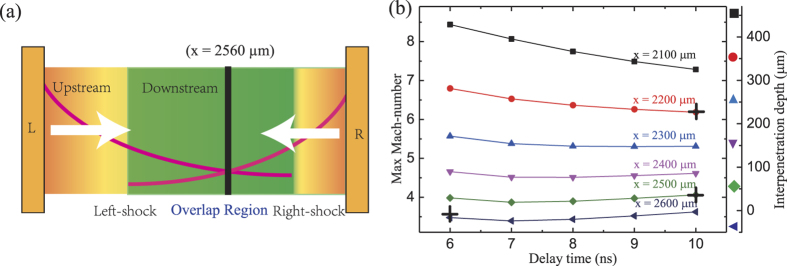
Theoretical analysis results. (**a**) The schematic diagram of interaction between CF. The pink-solid-lines on both sides represent the density distribution, which is obtained at 3 ns in the experiment. The black-solid-line represents the initial interaction position. Two shocks will form in the overlap region and propagate towards ±*x*. rections. (**b**) The theoretical prediction of the time of evolution of the Mach number at the different positions *x*. The black crosses represent the estimated Mach number using 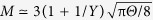.

Sorasio *et al*.’s theory^14^ has shown that stable high Mach number of electrostatic shock can form in CF with arbitrary density and temperature. The Mach number can be expressed as 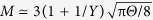, where *Y* and Θ are the density and temperature ratios between CF. Figure 6(a) shows the schematic diagram of interaction between CF. Each flow has the different density distributions and the same sound velocity, as obtained at 3 ns in the experiment. Initially, both flows meet near to the midplane (*x* = 2560 μm), where both flow parameters (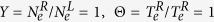) are the same. After both flows interpenetrating each other, the initial density ratio between both flows (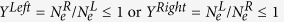) will decrease with the increase of the interpenetration depth (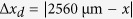). It is caused by the laser-ablation upstream with a quasi-exponent-form density distribution. Figure 6(b) shows the theoretical prediction of the time of evolution of the Mach number at the different positions *x (x* ≤ 2600 μm). Here we set *Θ* to be unity, since the sound velocity on both sides is almost same as shown in Fig. 2. The density ratio, *Y,* can be calculated according to the density distribution function. The Mach number obtained at 6 ns and 10 ns are estimated as 3.5 and 5/4 (marked by the black cross), respectively. It is in good agreement with experimental estimation. Additionally, one can find that the Mach number increases with the interpenetration depth 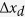. It means that the strength of the shocks will be enhanced by the decreasing initial (undisturbed) density ratio (*Y*) during the evolution. This positive feedback can offset the Mach number decay process. This is the reason why the shocks in the experiment stable for such a long time (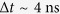) probably.

According to above discussions, we can conclude that the Mach number of the shocks in the symmetrical CF system cannot be infinite, because of the limited density ratio which is dependent on the initial exponential distribution of the laser-ablation plasma. In order to obtained high Mach number shocks, the temperature difference (*Θ*) should be induced. The unsymmetrical CF system should be a good choice, which can be generated by the unsymmetrical-laser ablation^12^,^13^, or by ablating different materials. The latter case is more similar to the actually astrophysical conditions that a shock forms at the interface between both clouds with different properties (density, temperature and component). Further experiments are needed to study the high Mach number shocks formation of those cases.

## Conclusions

In conclusion, formation of a pair of stable collisionless shocks and their propagation in opposite directions are firstly observed using laser directly produced CF in the experiment. The theoretical analysis and PIC simulations show that a bipolar electrostatic field excites the shocks formation and thermal pressure gradient driven the shocks evolution. Comparison of the experimental results with the PIC results shows that the positive feedback of shocks enhanced by the density ratio between both flows can offset the shock decay process. Therefore, the shocks can survive for a longer time. In additional, from the experimental data we find that the Mach number of the shocks in the symmetry case mainly depend on the initial density ratio between the upstream and the downstream.

## Methods

### Experimental setup

The experimental setup is schematically shown in Fig. 1. A pair of opposing Cu foils, separated by 4.5 mm (*L*), was used as the shock targets. The eight driven laser beams were symmetrically divided into two bunches, which were simultaneously focused on the facing surfaces of the foils with a focal spot diameter of 150 μm. The expanding plasma flows interacted with each other near to the midplane between the foils. The ninth laser beam with a wavelength of 527 nm and duration of 30 ps, transversely passing through the interaction region, was used as a probe for the optical diagnostics, including Nomarski interferometer, shadowgraphy and Faraday rotation. The time evolution of the counter-streaming flows is obtained by changing the delay time between the main beam and the probe beam.

### PIC simulations

A1D3V PIC simulation code^34^ has been performed to verify the formation mechanism of the shocks. Two identical plasma flows are counter-propagating. Each flow is with electron density of 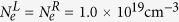, electron temperature of 400 eV (ion temperature of 140 eV) and flow velocity of 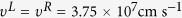. The ratio of ion mass to electron mass is 1836. Initially (*t* = 0), the right-moving flow occupies the domain −2250 μm ≤ *x* ≤ 0 μm (−1320 ≤ *x*/*λ*_*e*_ ≤ 0), and the left-moving flow occupies the domain 0 μm ≤ *x* ≤ 2250 μm (0 ≤ *x*/*λ*_*e*_ ≤ 1320). The length (*L*) of simulation box is 4500 μm, which is resolved by 225000 cells.

## Additional Information

**How to cite this article:** Yuan, D. *et al*. Formation and evolution of a pair of collisionless shocks in counter-streaming flows. *Sci. Rep.* **7**, 42915; doi: 10.1038/srep42915 (2017).

**Publisher's note:** Springer Nature remains neutral with regard to jurisdictional claims in published maps and institutional affiliations.
